# *Cysteine dioxygenase type 1 (CDO1)* gene promoter methylation during the adenoma-carcinoma sequence in colorectal cancer

**DOI:** 10.1371/journal.pone.0194785

**Published:** 2018-05-10

**Authors:** Keita Kojima, Takatoshi Nakamura, Makoto Ohbu, Hiroshi Katoh, Yosuke Ooizumi, Kazuharu Igarashi, Satoru Ishii, Toshimichi Tanaka, Keigo Yokoi, Nobuyuki Nishizawa, Kazuko Yokota, Yoshimasa Kosaka, Takeo Sato, Masahiko Watanabe, Keishi Yamashita

**Affiliations:** 1 Department of Surgery, Kitasato University School of Medicine, Minami-ku, Sagamihara, Kanagawa, Japan; 2 Department of Pathology, Kitasato University School of Allied Health Sciences, Minami-ku, Sagamihara, Kanagawa, Japan; 3 Division of Advanced Surgical Oncology, Department of Research and Development Center for New Medical Frontiers, Minami-ku, Sagamihara, Kanagawa, Japan; National Cancer Center, JAPAN

## Abstract

**Background:**

Progression of colorectal cancer (CRC) has been explained by genomic abnormalities along with the adenoma-carcinoma sequence theory (ACS). The aim of our study is to elucidate whether the promoter DNA methylation of the cancer-specific methylation gene, *cysteine dioxygenase 1* (*CDO1*), contributes to the carcinogenic process in CRC.

**Methods:**

The study group comprised 107 patients with CRC who underwent surgical resection and 90 adenomas treated with endoscopic resection in the Kitasato University Hospital in 2000. We analyzed the extent of methylation in each tissue using quantitative TaqMan methylation-specific PCR for *CDO1*.

**Results:**

The methylation level increased along with the ACS process (*p* < 0.0001), and statistically significant differences were found between normal-appearing mucosa (NAM) and low-grade adenoma (*p* < 0.0001), and between low-grade adenoma and high-grade adenoma (*p* = 0.01), but not between high-grade adenoma and cancer with no liver metastasis. Furthermore, primary CRC cancers with liver metastasis harbored significantly higher methylation of *CDO1* than those without liver metastasis (*p* = 0.02). As a result, the area under the curve by *CDO1* promoter methylation was 0.96, 0.80, and 0.67 to discriminate cancer from NAM, low-grade adenoma from NAM, and low-grade adenoma from high-grade adenoma, respectively.

**Conclusions:**

*CDO1* methylation accumulates during the ACS process, and consistently contributes to CRC progression.

## Introduction

Colorectal cancer (CRC) is a major cause of cancer deaths in Western countries [1]. Similarly, in Japan, CRC was the second most common cause of death from cancer in 2014 [2]. CRC is caused by genetic abnormalities such as genetic mutations or deletions, and accumulation of epigenetic abnormalities such as methylation of DNA. So far, two oncogenic pathways have mainly been proposed in CRC. One is the adenoma-carcinoma sequence (ACS): adenoma occurs first, and subsequently, cancer occurs in the adenoma with an increase in adenoma [3, 4]. The other is de novo carcinogenesis: cancer directly occurs in normal colorectal mucosa without adenoma [5]. ACS has been internationally well recognized, and in 1988, Vogelstein et al. proposed a multi-stage carcinogenesis model that conforms to ACS [6]. The model was as follows: as a result of multiple genetic changes in the adenoma, adenoma advances to carcinoma in situ and then to invasive carcinoma. So far, not only genetic abnormalities but also epigenetic abnormalities involved in ACS have been reported [7].

As one type of epigenetic abnormality associated with CRC, we have reported the aberrant methylation of *cysteine dioxygenase type 1* (*CDO1*). *CDO1* is a methylation-specific gene in human cancer that was identified by a pharmacological unmasking microarray [8, 9]. *CDO1* plays a role as a tumor suppressor gene and as a methylation-specific gene in human cancer. Methylation of the *CDO1* promoter region has been found in esophageal cancer [8, 10], gastric cancer [8], colorectal cancer [8], cholangiocarcinoma [11], lung cancer [8, 12], breast cancer [8], bladder cancer [8], prostate cancer [13], endometrial cancer [14], and hepatitis B virus-related hepatocellular carcinoma (HBV-related HCC) [15]. The degree of malignancy or cancer progression with methylation has been reported for some cancers: gallbladder cancer [16], Barrett esophagus cancer [17], esophageal squamous cell carcinoma [10], and HBV-related HCC [15]. In breast cancer [18], gallbladder cancer [16], renal clear-cell cancer [19], esophageal squamous cell carcinoma [10], and lung cancer [20], methylation abnormalities in *CDO1* have been reported as a prognostic factor. Thus, methylation abnormalities in *CDO1* reflect not only the changes that accumulate with progression but also the degree of malignancy. Aiming at practical applications in which *CDO1* methylation may serve as a biomarker, research on lung cancer [21], gastric cancer [22], colon cancer [23], HBV-related HCC [15], and cholangiocarcinoma [24] has been conducted.

*CDO1* encodes a non-heme iron enzyme that converts cysteine to cysteine sulfinic acid [8, 25, 26]. Cysteine sulfinic acid suppresses H^+^ efflux from the mitochondria to intracellular compartments and induces the maintenance of mitochondrial membrane potential [27]. On the other hand, CDO1 suppresses the production of glutathione from cysteine and induces reactive oxygen species generation, subsequently promoting apoptosis [28].

Although *CDO1* methylation is clearly associated with carcinogenesis, at what stage of carcinogenesis aberrant methylation of *CDO1* occurs is unclear. Thus, the aim of our study was to elucidate how *CDO1* methylation contributes to carcinogenesis during the carcinogenic process of CRC, and to clarify the clinical significance of *CDO1* methylation in primary CRC.

Epi proColon^®^ is a representative clinical application of colon cancer diagnostic technology based on epigenomics. Epi proColon^®^, which was approved by the Food and Drug Administration in 2016, is based on methylation of *SEPT9* in serum. This test has proven to be useful as a biomarker for CRC in multiple papers [29, 30]. Therefore, the possibility of clinical application of methylation abnormalities of *CDO1* was examined by comparing the results with *SEPT9*, which is an existing leading biomarker.

## Materials and methods

### Patients and tissue samples

The study group comprised 107 patients with CRC who underwent surgical resection in Kitasato University Hospital from January 1, 2000 through December 31, 2000. The details of the patients are shown in Table 1. Specimens were cancerous tissue and corresponding normal-appearing mucosa (NAM) without pathological atypia. We previously investigated tissues of normal from cancer patients and normal form normal patients, and normal from cancer patients harbored higher methylation as compared to the normal from normal patients [8]. No patients received preoperative chemoradiation therapy. Forty-one patients received postoperative chemotherapy.

**Table 1:** Relationship with TaqMeth V of CDO1 and clinicopathological factors at cancer tissue.

Clinicopathological factors	Compare items	n	Average of TaqMeth V	*P*-value	
Age		107	---	0.006	(R^2^ = 0.070)
Gender	Male	65	40.6 ± 22.9	0.73	
	Female	42	39.1 ± 20.7		
Histological type	Differentiated type	99	41.3 ± 22.0	0.03	
	Undifferentiated type	8	24.7 ± 16.4		
Tumor location	Colon	60	38.7 ± 20.9	0.49	
	Rectum	47	41.7 ± 23.4		
Tumor diameter (cm)		107	---	0.04	(R^2^ = 0.038)
Liver metastasis	Negative	80	37.2 ± 21.7	0.02	
	Positive	27	48.6 ± 20.8		
Depth of tumor invasion	~sm	13	29.8 ± 20.0	0.07	
	mp~	94	41.5 ± 22.0		
Lymph node metastasis	Negative	60	37.6 ± 21.7	0.19	
	Positive	47	43.2 ± 22.2		
Distant metastasis	Negative	88	38.7 ± 21.7	0.16	
	Positive	19	46.4 ± 22.9		
pStage	0	4	25.4 ± 11.7	0.45	
	I	23	35.6 ± 20.7		
	II	31	39.2 ± 23.3		
	III	30	42.2 ± 21.5		
	IV	19	46.4 ± 22.9		
Dukes classification	A	27	34.1 ± 23.3	0.30	
	B	31	39.2 ± 23.3		
	C	30	42.2 ± 21.5		
	D	19	46.4 ± 22.9		
Dukes classification	A and B	58	36.8 ± 21.7	0.10	
	C and D	49	43.9 ± 21.9		
Lymphatic invasion	Negative	96	30.2 ± 22.0	0.11	
	Positive	11	41.2 ± 21.8		
Venous invasion	Negative	95	32.5 ± 19.4	0.07	
	Positive	22	42.0 ± 22.3		
Infiltrative pattern	a	5	24.4 ± 23.2	0.75	
	b	91	41.7 ± 21.0		
	c	6	41.2 ± 27.9		

**Fig 1:**
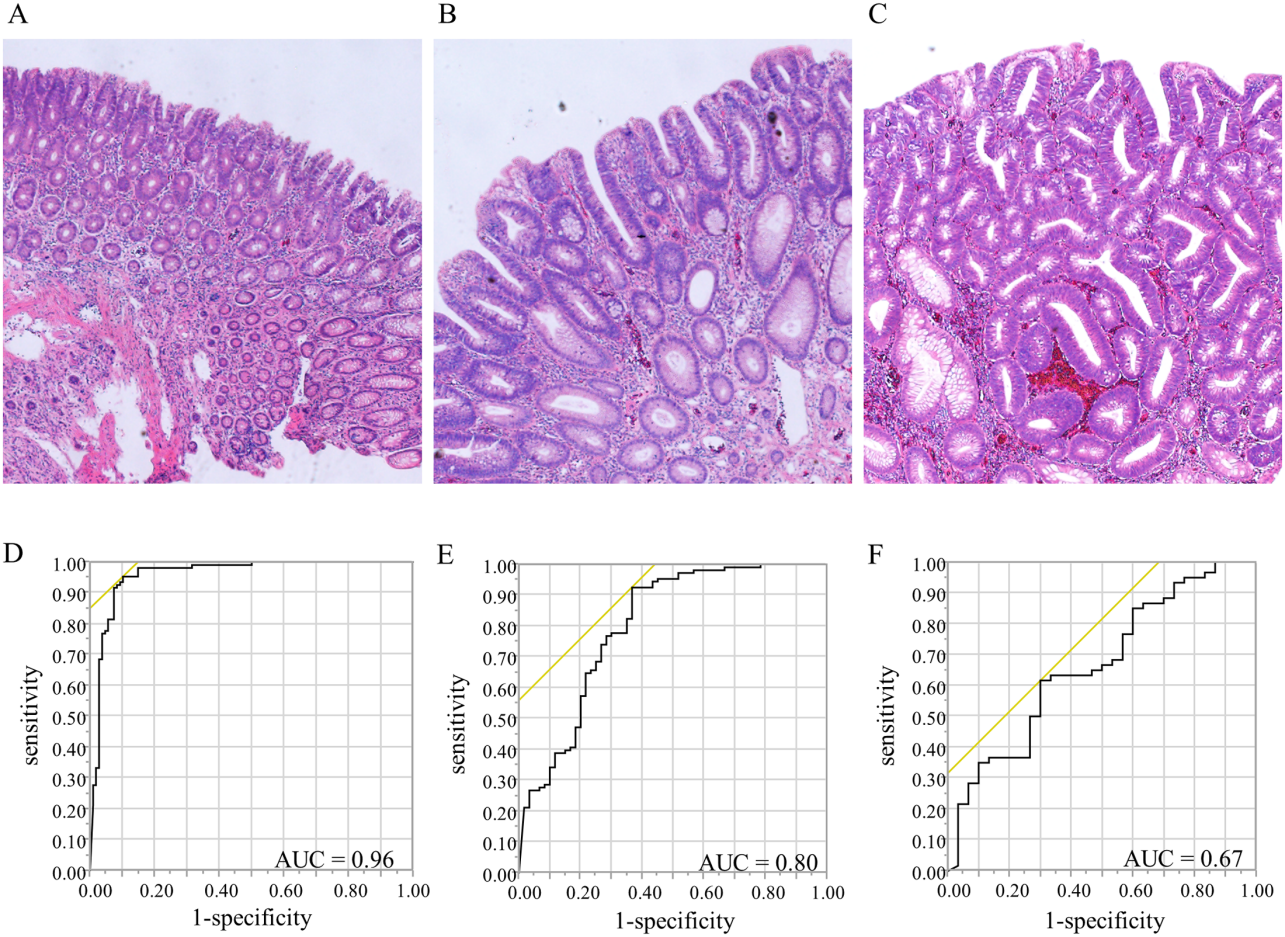
Standard light microscopy findings on hematoxylin-eosin-stained specimens of colorectal adenoma (40×, magnification) and ROC curves for distinguishing between the two tissues. A: mild atypia. B: moderate atypia. C: severe atypia. D: ROC curve of *CDO1* promoter methylation for distinguishing between cancer tissue and NAM. When the cut-off value was 15.6, the AUC was 0.96, sensitivity was 95%, and specificity was 90%. E: ROC curve for distinguishing between low-grade adenoma and NAM. F: ROC curve for distinguishing between low-grade adenoma and high-grade adenoma.

To understand the mechanism of *CDO1* methylation in the carcinogenesis pathway, 90 adenomas that were endoscopically resected in 2000 were also included. Adenoma is classified into three categories, mild atypia, moderate atypia, and severe atypia, including cellular atypism and structural atypism according to the 7th general rules for clinical and pathological studies on cancer of the colon, rectum, and anus [31]. Further, mild and moderate atypia are classified into low-grade adenoma, and severe atypia is classified into high-grade adenoma [32]. We performed our investigation in accordance with this classification (Fig 1A, 1B and 1C). Adenomas consisted of 30 with mild atypia, 30 with moderate atypia, and 30 with severe atypia.

Clinicopathological factors of the evaluation target included age, gender, histological type (differentiated types are tubular adenocarcinoma and papillary adenocarcinoma; undifferentiated types are poorly differentiated adenocarcinoma and mucinous adenocarcinoma), tumor location, tumor diameter, liver metastasis (including the course of preoperative and postoperative periods), pathological depth of tumor invasion (T), lymph node metastasis (N), distant metastasis (M), staging classification, lymphatic invasion (ly), and vascular invasion (v) according to the 7th edition of the American Joint Committee on Cancer/International Union Against Cancer (7th UICC) staging system, Dukes classification, and the infiltrative growth pattern (INF), which was judged according to the 8th Japanese classification of colorectal carcinoma [32].

We used formalin-fixed and paraffin-embedded (FFPE) samples. Unlike fresh frozen samples, FFPE samples may exhibit deterioration in the quality of DNA [33]. However, verification of methylation analysis using FFPE specimens has been done, and the usefulness has been confirmed [34]. For this reason, methylation analysis was also carried out using FFPE samples in this study. The present study was approved by the Ethics Committee of Kitasato University. The approval number is B17-004. Clinical investigation have been conducted according to the principles expressed in the Declaration of Helsinki. All tissue samples were collected at the Kitasato University Hospital, all patients had agreed to the use of their pathological specimens and written consent was obtained from all patients before sample collection.

### Cell lines

The CRC cell line DLD1 was kindly provided from the Cell Resource Center for Biomedical Research Institute of Development, Aging and Cancer, Tohoku University (Sendai, Japan). The CRC cell line HCT116 and the HCC cell line HepG2 were purchased from RIKEN BioResource Centre (Ibaraki, Japan).

DLD1 cells and HCT116 cells were grown in RPMI 1640 medium (GIBCO, Carlsbad, CA), and HepG2 cells were grown in DMEM (GIBCO). All media contained 10% fetal bovine serum and penicillin-streptomycin (GIBCO).

### Reverse transcriptase PCR

Total RNA from cell lines was extracted using the RNeasy Mini Kit (Qiagen, Hilden, Germany). cDNA was synthesized from RNA using SuperScript III reverse transcriptase (Invitrogen) and Oligo (dT) primers (Invitrogen, Carlsbad, CA). The obtained cDNA was used for RT–PCR, which was carried out using Platinum Taq DNA Polymerase (Invitrogen) according to the manufacturer’s protocol. The PCR conditions were: 5 min at 95 °C followed by 30 cycles of 95 °C for 1 min, 58 °C for 30 sec, and 72 °C for 30 sec, and a subsequent final incubation at 72 °C for 5 min. Primer sequences are shown in S1 Table. The positive control for expression of *CDO1* was DLD1 cells, and the negative control was HepG2 cells. These controls for *CDO1* were selected based on our previous reports [8, 16, 18].

### DNA purified from tissue and bisulfite treatment of DNA

Tissue sections were sharply dissected on hematoxylin and eosin-stained slides. Genomic DNA was subsequently extracted using a QIAamp DNA FFPE Kit (Qiagen Sciences, Hilden, Germany). Bisulfite treatment was carried out using an EZ DNA Methylation-Gold^™^ Kit (Zymo Research, Orange, CA).

### Quantitative methylation-specific PCR (Q-MSP)

Quantitative TaqMan MSP was performed using iQ Supermix (Bio-Rad, Hercules, CA) in triplicate on the iCycler iQTM Real-Time PCR Detection system (Bio-Rad). Q-MSP was done at 95 °C for 3 min, followed by 40 cycles at 95 °C for 20 sec, annealing temperature (60 °C) for 30 sec, and 72 °C for 30 sec in a 25-μL reaction volume containing 1 μL bisulfite-treated genomic DNA, 300 nmol/L each primer, 200 nmol/L fluorescent probe, and 12.5 μL iQ^™^ Supermix. Sequences of primers and probes are provided in S1 Table.

The positive control for methylation was DLD1 cells, and the negative control was HepG2 cells. These controls for *CDO1* were selected based on our previous reports [8, 23]. Specifically, in the cloned sequence, 95% of the total methylation sites were methylated in DLD1 cells, and only 4% in HepG2 cells were methylated. Therefore, we used these as positive and negative controls in *CDO1* methylation analysis.

In addition, the *SEPT9* control was determined by the bisulfite cloning sequence in the primer region as reported in CRC [30]. The methylation value (TaqMeth V) was defined as the quantity of fluorescence intensity derived from promoter amplification of the positive control gene divided by the fluorescence intensity from β-actin and then multiplied by 100 [35].

### Bisulfite sequencing analysis

The Q-MSP primer for *SEPT9* was cited as reported in a previous paper [30]. Primers for bisulfite sequencing analysis were prepared to include the analysis region of the Q-MSP primer for *SEPT9* (S1 Table). Q-MSP of *SEPT9* was performed on cell lines. Bisulfite sequencing analysis was performed with DLD1 cells, which showed a high TaqMeth V and HepG2 cells, which showed a TaqMeth V = 0. For details of bisulfite sequencing, refer to the previous report [23].

### Plasmid transfection

A full-length cDNA for *CDO1* was isolated and cloned into the pcDNA3.1 myc-His C expression vector (Invitrogen). HCT116 cells were transfected with *CDO1* plasmid vectors using Lipofectamine 2000 (Invitrogen) in Opti-MEM (Invitrogen) as per the manufacturer’s instructions.

### Anchorage-independent colony formation assay

The anchorage-independent colony formation assay was performed as follows. In a six-well plate, 0.72% agarose (Bacto Agar; Becton, Dickinson and Company, Franklin Lakes, NJ) was placed on the bottom. Top agar was made with agarose mixed with 1 × 10^5^ HCT116 cells transfected with *CDO1*. After 2 weeks of culture, colonies with more than 100 cells were counted in 10 fields of view. The experiment was conducted twice.

### Immunostaining for CDO1

FFPE tissue blocks were cut into thin sections (4 μm thick) that were then deparaffinized with xylene and dehydrated through a stepwise series of ethanol. For antigen activation, samples were immersed in pH 6 citrate buffer and boiled in a microwave for 15 min. The sections were then incubated in 3% aqueous hydrogen peroxide for 15 min to inactivate endogenous peroxidases. The sections were incubated with primary rabbit anti-CDO1 polyclonal antibody (12589-1-AP) (proteintech, Rosemont, IL; 1:100) overnight at 4 °C. The secondary antibody reaction was performed using the Histofine Simple Stain MAX-PO(MULTI) kit (Nichirei, Tokyo, Japan) according to the manufacturer’s protocol. Color was developed by incubating with ImmPACT DAB (Vector Laboratories, Inc., Burlingame, CA) for 5 min. Mayer’s Hematoxylin Solution was used to stain nuclei. Immunostaining was scored as follows: Score 0 indicates not stained, Score 1 indicates staining that is interspersed or light, and Score 2 indicates diffuse, deep staining. Evaluation was performed in two arbitrary fields of view of each specimen.

### Statistical analysis

Analysis of the relationship between *CDO1* TaqMeth V and clinicopathological factors was done with Student’s t-test, Mann-Whitney’s U test, Tukey’s honestly significant difference test, variance, and Kruskal-Wallis test, as appropriate. The test of homoscedasticity was performed with the F test and Levene test.

Estimated cumulative 5-year relapse-free survival (RFS) and overall survival (OS) were calculated with the Kaplan-Meier method, and statistical differences were tested with the log rank test. RFS and OS were measured from the date of surgery to the date of events or the last follow-up. Variables suggested to be prognostic factors in univariate analysis were subjected to multivariate analysis using a Cox proportional-hazards regression model. *P* < 0.05 was considered statistically significant. All statistical analyses were performed with SAS software package JMP, version 11 (SAS Institute Inc., Cary, NC).

## Results

### DNA methylation of the *CDO1* and *SEPT9* promoter regions in CRC tissue

First, we measured the degree of excessive methylation of the *CDO1* promoter DNA regions in cancerous tissue and corresponding NAM in primary CRC. The median TaqMeth V of *CDO1* in cancerous tissue was 34.8 (range, 0 to 100.3), and in NAM was 4.3 (range, 0 to 35.5). The TaqMeth V for each methylation level was significantly higher in cancerous tissue than in NAM (*p* < 0.0001).

Based on these results, we determined the cut-off value of *CDO1* TaqMeth V that discriminates cancerous tissue from NAM using receiving-operating characteristic (ROC) curves. The most optimal cut-off value was 15.6 [area under the curve (AUC), 0.96; sensitivity, 95%; specificity, 90%] (Fig 1D).

For *SEPT9*, cancer-specific methylation abnormalities were observed, similar to a previous report [cancer tissue TaqMeth V = 3.17 (0–17.74), NAM TaqMeth V = 0 (0–4.25), *p* < 0.0001] [30]. The cut-off value that could distinguish colon cancer tissue from NAM was 0.06 (sensitivity 94.4%, specificity 95.3%, AUC = 0.96) (S1A Fig). The results of bisulfite sequencing of *SEPT9* showed hypermethylation in DLD1 cells (99.2%) and hypomethylation in HepG2 (0%) cells (S2 Fig).

### Relationship between *CDO1* and *SEPT9* TaqMeth V and clinicopathological factors

**Fig 2:**
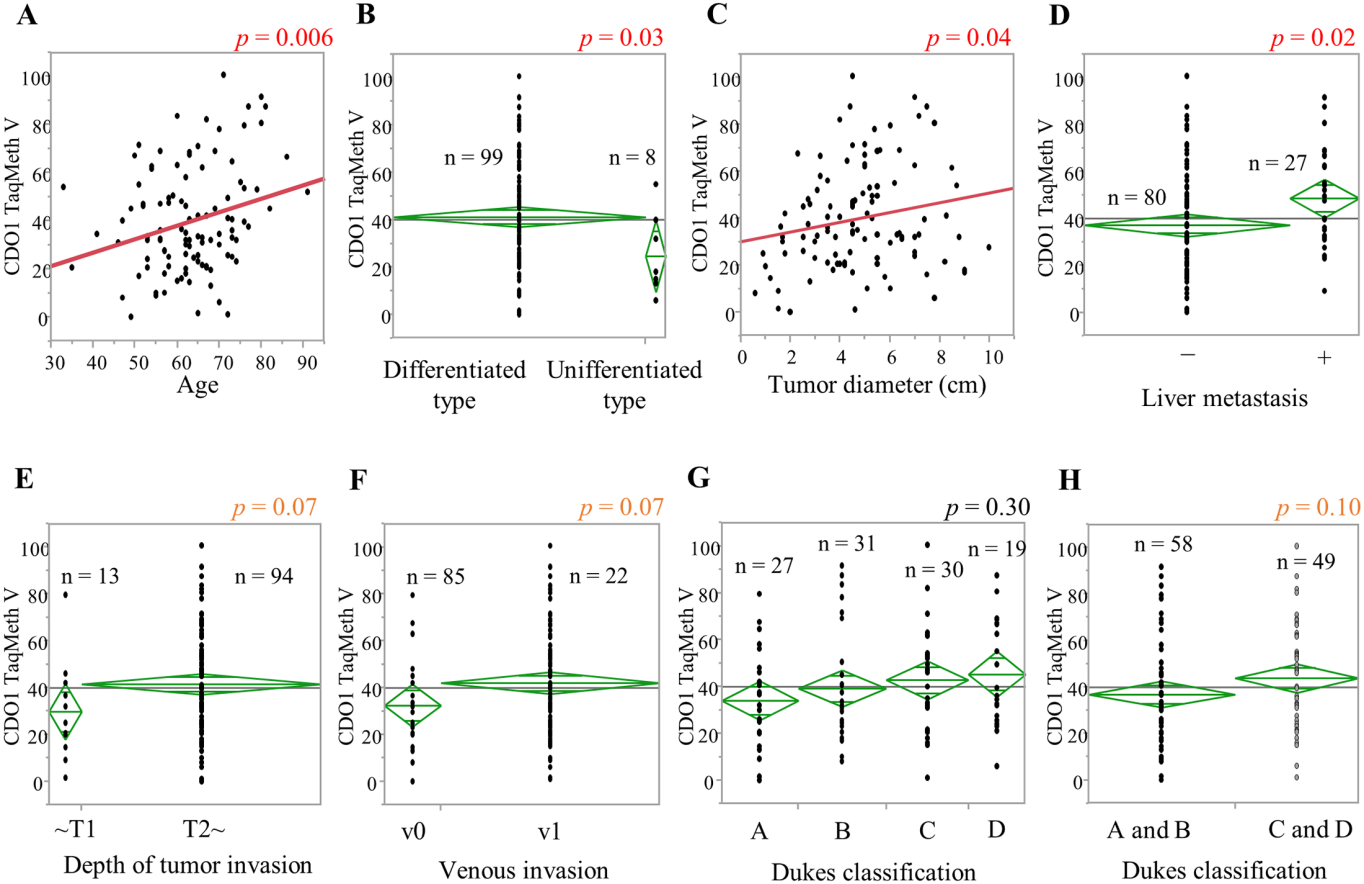
Associations of clinicopathological factors with *CDO1* TaqMeth V in cancerous tissue. The relationship between *CDO1* and A: age, B: histological type, C: tumor diameter, D: liver metastasis, E: pT, F: v, G and H: Dukes classification.

Next, we examined the clinicopathological characteristics of the *CDO1* promoter DNA TaqMeth V in primary CRC tissue. The results are shown in Table 1 and Fig 2. *CDO1* TaqMeth V showed a significant difference according to age (*p* = 0.006; R^2^ = 0.070), histological type (*p* = 0.03), tumor diameter (*p* = 0.04; R^2^ = 0.038), and liver metastasis (*p* = 0.02). In addition, the difference was marginally significant for pT (*p* = 0.07), Dukes classification (*p* = 0.10), and v (*p* = 0.07).

No significant difference was found between TaqMeth V of *SEPT9* and clinicopathological factors in CRC tissue. In particular, for *CDO1*, a significant difference was observed in the presence or absence of liver metastasis, but for *SEPT9*, no significant difference was found (*p* = 0.54).

### Tumor-suppressive activity after transfection with *CDO1*

**Fig 3:**
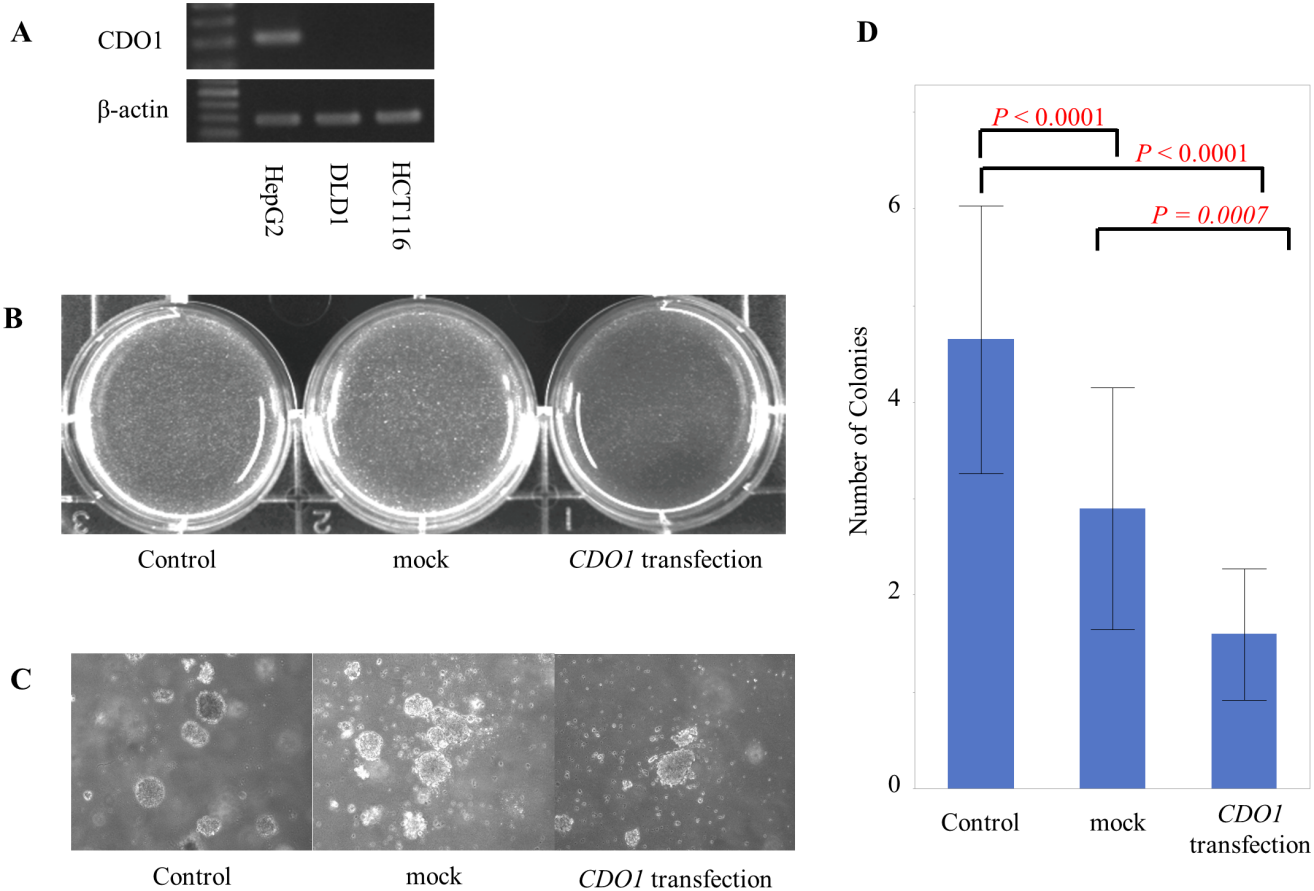
Results of the anchorage-independent colony formation assay. A: Expression of *CDO1* cDNA by RT-PCR. The positive control was HepG2 cells, and the negative control was DLD1 cells [8, 16, 18]. In HCT116 cells, expression of *CDO1* was not observed. B: Control, not transfected; mock, mock treatment; *CDO1* transfection, *CDO1* cells transfected with pcDNA 3.1-CDO1. The colonies were photographed under UV after staining with ethidium bromide staining. The number of colonies was small in the cells transfected with *CDO1*. C: Colony formation of each experimental condition. Colonies were photographed under phase-contrast microscope. The image is magnified 100 times. D: The numbers of colonies are shown. Cell proliferation by HCT116 cells was suppressed by transfection of *CDO1*.

Accumulation of methylation abnormalities in *CDO1* may be involved in liver metastasis, and thus, we evaluated regulation of cell proliferation by *CDO1* with the anchorage-independent colony formation assay. HCT116 cells, which were confirmed with RT-PCR to not express *CDO1*, were transfected with *CDO1*. Colonization was significantly reduced in cells transfected with *CDO1* compared to mock-transfected cells (*p* = 0.0007) (Fig 3).

### CDO1 immunostaining in colorectal tissue

**Fig 4:**
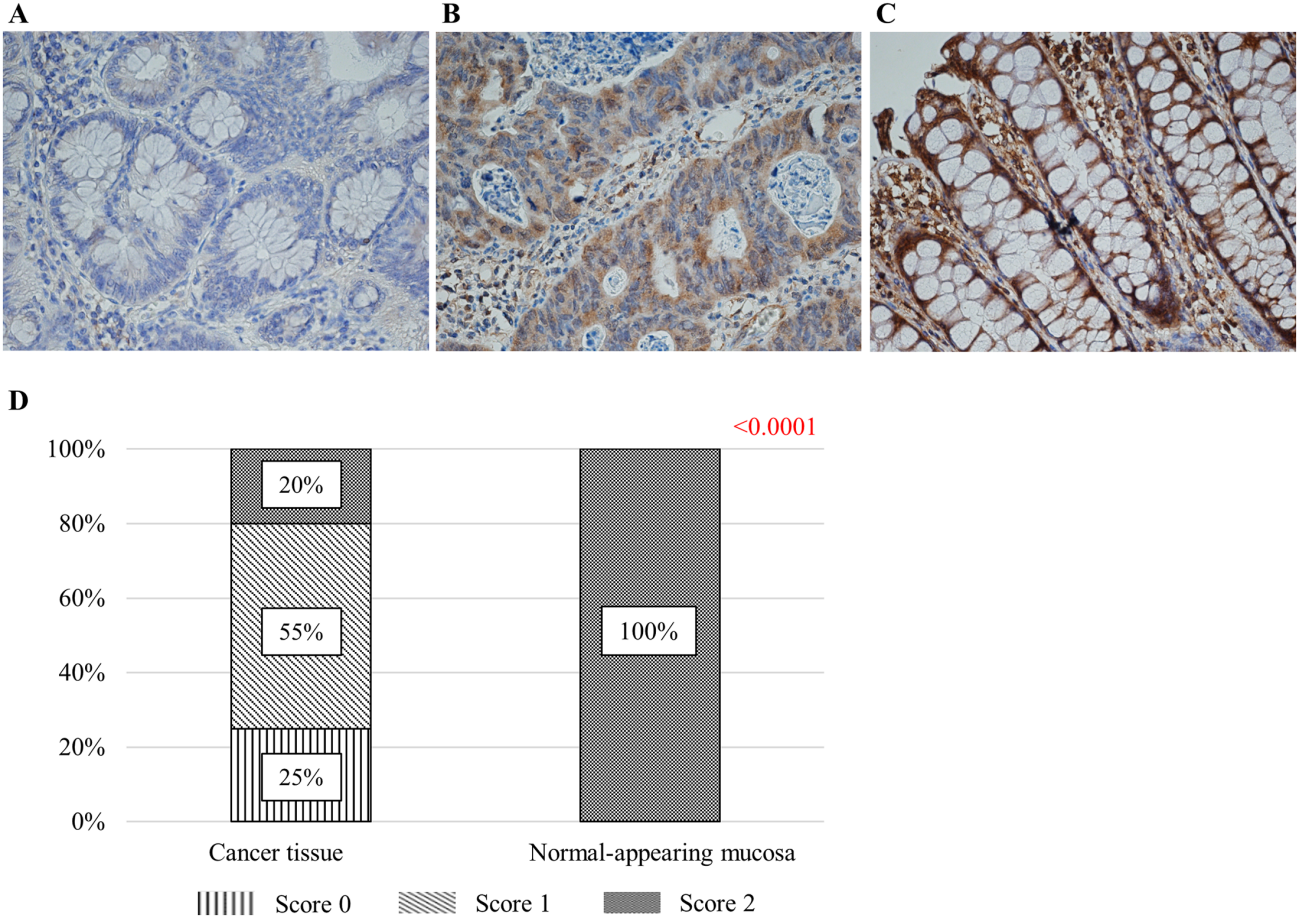
Results of CDO1 immunostaining. A: IHC score = 0. B: IHC score = 1. C: IHC score = 2. D: Results of IHC scoring in cancer tissue and NAM are shown. Intense staining was observed in NAM, and less intense staining was seen in cancer tissues. A significant difference in scoring was seen between the groups.

Next, to evaluate the relationship between methylation of the promoter region and protein expression, immunostaining was carried out using 10 hypomethylated specimens (average TaqMeth V = 0) and 10 hypermethylated specimens (average TaqMeth V = 75.4). Immunostaining for CDO1 stained the cytoplasm of gland epithelial cells and cancer cells. Scoring results were as follows. In the low methylation group, all cases received a score of 2. In the hypermethylated group, 25% were score 0, 55% were score 1, and 20% were score 2. Significantly high expression of CDO1 was observed in the hypomethylated group (*p* < 0.0001) (Fig 4).

### Prognostic analysis in patients with CRC

**Fig 5:**
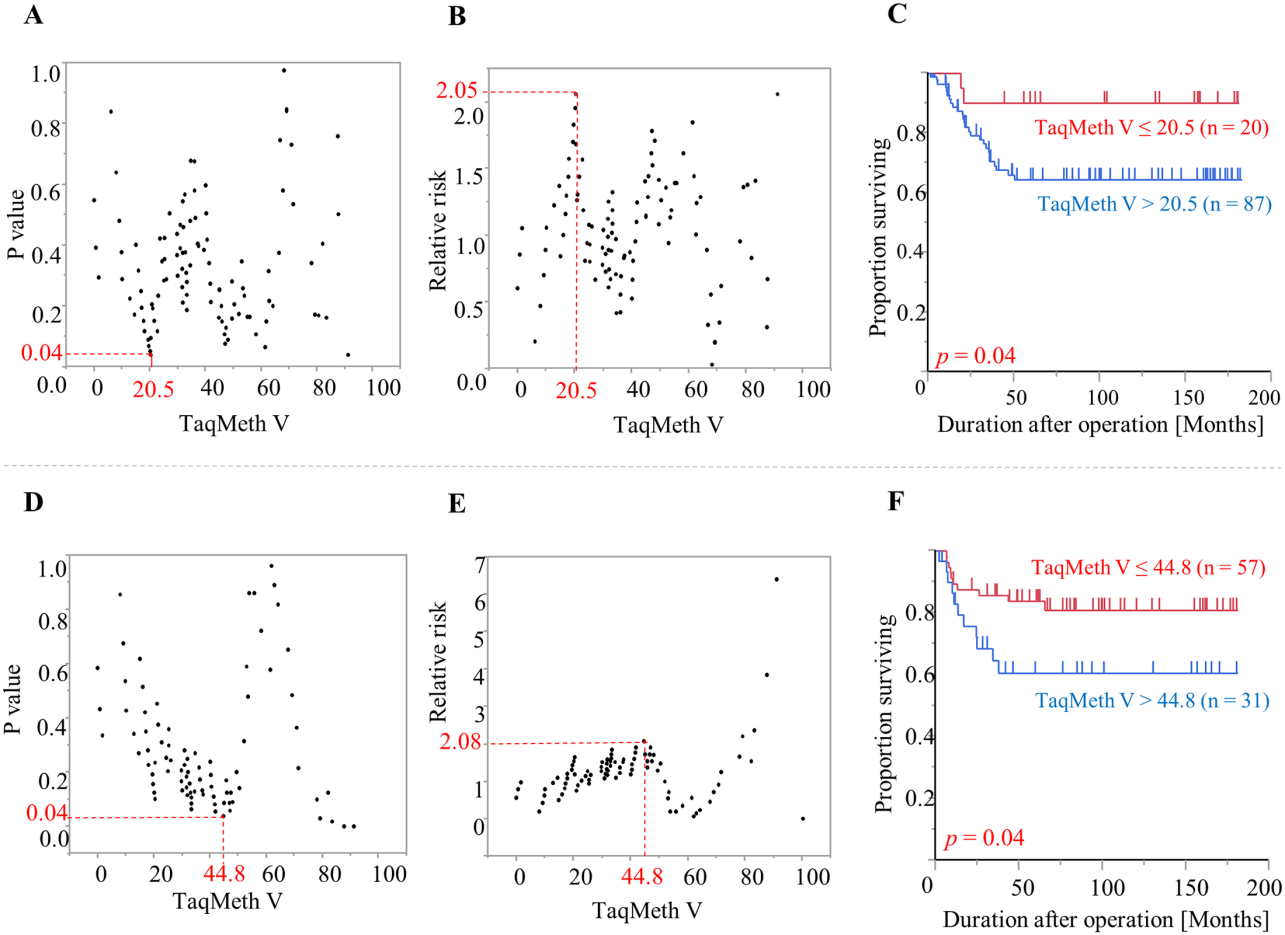
Log-rank plot analysis and Kaplan-Meier survival curves for OS and RFS in CRC patients according to *CDO1* TaqMeth V. A, B: Log-rank plot analysis for OS. C: Kaplan-Meier survival curves for OS comparing CRC patients with *CDO1* TaqMeth V equal to or below 20.5 and those with *CDO1* TaqMeth V over 20.5. D, E: Log-rank plot analysis for RFS. F: Kaplan-Meier survival curves for RFS comparing CRC patients with *CDO1* TaqMeth V equal to or below 44.8 and those with *CDO1* TaqMeth V over 44.8.

Using a log-rank plot analysis, which was performed to calculate the optimal cut-off values for OS and RFS, we examined whether *CDO1* TaqMeth V in CRC can potentially be used as a prognostic factor. The optimal cut-off value for *CDO1* TaqMeth V was 20.5 for OS (*p* = 0.04; relative risk, 2.05) and 44.8 for RFS (*p* = 0.04; relative risk, 2.08) (Fig 5).

The CRC patients were divided into two groups [*CDO1* high TaqMeth V group (TaqMeth V > cut-off value); *CDO1* low TaqMeth V group (TaqMeth V ≤ cut-off value)]. We then performed univariate prognostic analysis of clinicopathological factors and the TaqMeth V high or low group for OS and RFS using a log-rank test. pT, pN, pM, pStage (Dukes classification), ly, v, INF, and *CDO1* TaqMeth V were prognostic factors in the univariate analysis for OS. Because pT, pN, and pM are included in pStage, pStage was used in subsequent analyses. Multivariate analysis was performed with a Cox proportional hazards model, including the significant prognostic factors confirmed in univariate analysis. The results showed that pStage was the only independent prognostic factor (Table 2).

**Table 2:** Univariate and multivariate prognostic analysis of clinicopathological factors for OS in colorectal cancer.

Clinicopathological parameters	Categories	account	Univariate analysis		Multivariate analysis		
			5year OS(%)	*p*-value	Hazard ratio	95%CI	*p*-value^※^
Age	≤ 63	56	67.9	0.50			
	63 <	51	72.4				
Gender	Male	65	74.3	0.37			
	Female	42	63.5				
Histological type	Differentiated type	99	71.7	0.41			
	Undifferentiated type	8	50.0				
Tumor location	Colon	60	76.9	0.13			
	Rectum	47	61.5				
Liver metastasis	Negative	80	84.7	<0.0001	1.62	0.55–4.66	0.37
	Positive	27	16.4				
Depth of tumor invasion (pathological)	~sm	13	100	0.02	---		
	mp~	94	65.0				
Lymph node metastasis (pathological)	Negative	60	96.2	<0.0001	---		
	Positive	47	33.1				
Distant metastasis (clinical)	Negative	88	80.6	<0.0001	---		
	Positive	19	7.7				
pStage (Dukes classification)	pStage 0—I (A)	27	100	<0.0001	1		<0.0001
	pStage II (B)	31	96.0		5.83×10^8^	0.07–6.93e^115^	
	pStage III (C)	30	49.1		7.81×e^9^	2.62–1.14e^77^	
	pStage IV (D)	19	7.1		2.65×e^10^	7.52–2.08e^63^	
Lymphatic permeation (ly)	ly0	11	100	0.03	0.21	3.4e^-158^ - -	1.0
	ly1	96	65.9				
Vascular permeation (v)	v0	22	100	0.002	5.41×10^8^	0.43 - -	0.18
	v1	85	61.8				
Infiltrative growth pattern (INF)	INF a, b	96	72.9	<0.0001	2.87	0.71–8.95	0.13
	INF c	6	0				
CDO1 TaqMeth Value	≤ 20.5	20	90.0	0.04	1.98	0.47–14.0	0.38
	20.5 <	87	64.4				

^※^Cox proportional-hazards model

Similarly, we performed prognostic analysis excluding liver metastasis as a factor from the subjects in pStage 0 to III for RFS. Tumor location, pT, pN, pStage (Dukes classification), v, INF, and *CDO1* TaqMeth V were significant prognostic factors for RFS. Multivariate prognostic analysis showed that pStage was the only independent prognostic factor (S2 Table).

### Aberrant *CDO1* promoter methylation in adenoma

The mean tumor diameter was 2.7 ± 1.2 mm for adenomas with mild atypia, 4.7 ± 2.9 mm for adenomas with moderate atypia, and 7.5 ± 4.3 mm for adenomas with severe atypia (*p* < 0.0001). The median TaqMeth V was 14.1 (range, 0 to 80.0) for mild atypia, 19.3 (range, 0.8 to 72.2) for moderate atypia, 25.5 (range, 0 to 183.6) for severe atypia, and 17.2 (range, 0 to 80.0) for low-grade adenoma. An analysis of the clinicopathological background factors in adenoma showed that *CDO1* TaqMeth V tended to increase in parallel with age (*p* = 0.006, R^2^ = 0.039).

**Fig 6:**
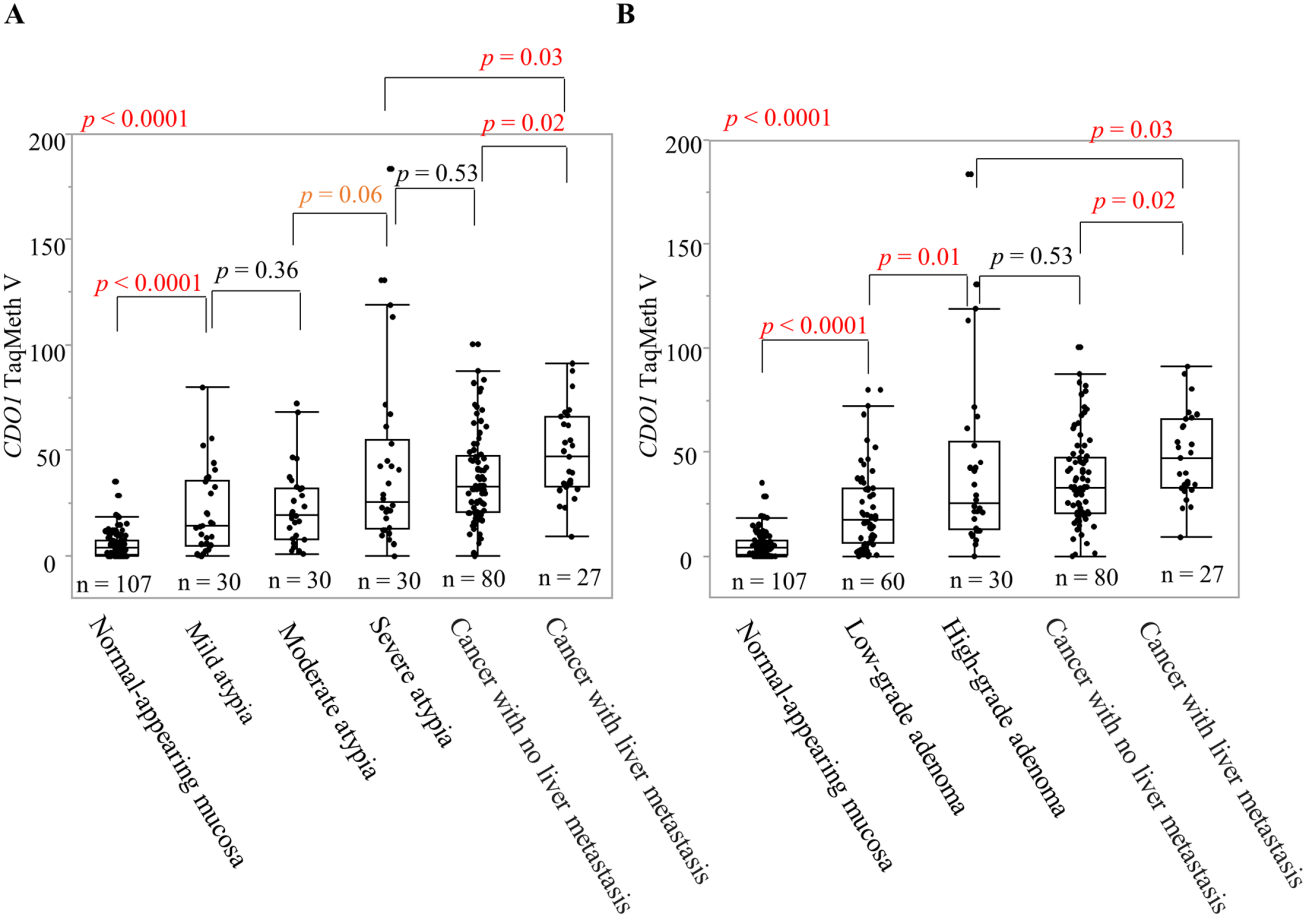
Analysis of *CDO1* TaqMeth V derived from NAM, adenoma, and cancerous tissue. A: Adenoma is classified into three categories: mild atypia, moderate atypia, and severe atypia. B: Adenomas were divided into low-grade adenoma and high-grade adenoma.

TaqMeth V was compared among the following five groups: NAM, mild atypia, moderate atypia, severe atypia, and cancer tissue (*p* < 0.0001) (Fig 6A). TaqMeth V differed significantly between NAM and mild atypia (*p* < 0.0001), between mild atypia and severe atypia (*p* = 0.01), and between moderate atypia and cancer tissue (*p* < 0.0001). TaqMeth V was slightly but not significantly higher in severe atypia than in moderate atypia (*p* = 0.06). We found no significant difference between mild atypia and moderate atypia (*p* = 0.36) or between cancer tissue and severe atypia (*p* = 0.22). In addition, the cancer tissues were divided into the presence or absence of liver metastasis, and TaqMeth V was compared. Although no significant difference was found between severe atypia and cancer tissue with no liver metastasis (*p* = 0.53), the TaqMeth V differed significantly between cancer tissue with liver metastasis and severe atypia (*p* = 0.03).

Next, the adenomas were divided into low-grade and high-grade adenomas based on the latest classification, and TaqMeth V was compared. TaqMeth V differed significantly between NAM and low-grade adenoma (*p* < 0.0001), between low-grade adenoma and high-grade adenoma (*p* = 0.01), and between low-grade adenoma and cancer tissue (*p* < 0.0001) (Fig 6B). In this way, TaqMeth V in NAM, adenoma, and cancer tissue tended to increase in parallel with the degree of atypia.

The cut-off *CDO1* TaqMeth V that could distinguish low-grade adenoma from NAM was determined with ROC curve analysis. The cut-off value was 13.0 (AUC, 0.80; sensitivity, 93%; specificity, 63%) (Fig 1E). The cut-off value that distinguished between low-grade adenoma and high-grade adenoma was 20.4 (AUC, 0.67; sensitivity, 62%; specificity, 70%) (Fig 1F).

Similarly, TaqMeth V of adenoma was evaluated for *SEPT9*. The median values of TaqMeth were 0 (0–2.41) for mild adenoma, 0.20 (0–8.62) for moderate adenoma, 0.02 (0–8.62) for low-grade adenoma, and 1.04 (0–7.29) for severe (high-grade) adenoma. As with *CDO1*, an elevation in methylation was observed with the progress of atypicality. For *CDO1*, a significant difference was observed between NAM and mild adenoma, but for *SEPT9*, no significant difference was found (*p* = 0.06). However, we did find a significant difference between NAM and low-grade adenoma (*p* < 0.0001). A significant difference was observed between low-grade adenoma and high-grade adenoma for *CDO1*, but no significant difference was observed for *SEPT9* (*p* = 0.10). For *CDO1*, no significant difference was found between high-grade adenoma and cancer tissue, but a significant difference was observed for *SEPT9* (*p* < 0.0001) (S3 Fig).

Similar to *CDO1*, a cut-off value of 0.01 (sensitivity 52%, specificity 95%, AUC = 0.73) was obtained using ROC curve analysis to distinguish between NAM and low-grade adenoma with *SEPT9*. In addition, a cut-off value of 0.34 (sensitivity 80%, specificity 67%, AUC = 0.73) was obtained to distinguish between low-grade adenoma and high-grade adenoma (S1 Fig).

## Discussion

Our study for the first time revealed the clinicopathological characteristics associated with methylation of *CDO1* in CRC tissue and clarified the relationship between *CDO1* methylation and ACS.

Methylation of DNA in cancer cells causes gene inactivation and contributes to carcinogenesis [36]. Our previous studies showed that *CDO1* methylation occurs more frequently in CRC tissue than in NAM [8]. However, the relationship between *CDO1* methylation and the clinicopathological characteristics of CRC has not been studied in detail.

Our findings indicated that TaqMeth V of *CDO1* in CRC was 34.8 in cancer tissue and 4.3 in NAM, and we estimated that the cut-off value of *CDO1* TaqMeth V that could be used to differentiate cancer tissue from NAM was 15.6. This cut-off value was similar to previously reported results (TaqMeth V of *CDO1* in CRC tissue, 38.4; TaqMeth V of *CDO1* in NAM, 5.0; cut-off value of *CDO1* TaqMeth V in CRC, 12.5) [8]. Although the specimens used in the two studies differed, reproducibility of *CDO1* methylation was confirmed. TaqMeth V was confirmed to be a highly reproducible and sensitive readout from Q-MSP [23]. In addition, reproducible results were obtained, even with FFPE samples.

Regarding the relationship between methylation abnormalities in *CDO1* and expression of CDO1 protein, our previous studies showed a significant relationship in gallbladder cancer [16]. In this study as well, we found that methylation of *CDO1* and expression are inversely correlated in CRC. The result of immunostaining resulted in suppression of expression in the hypermethylated group. Strong expression of protein was observed in all hypomethylated groups, and the level of staining decreased while showing heterogeneous staining among cells in a tumor. These findings suggest that methylation may also be functionally involved in CRC phenotypes in clinical samples. In addition, various staining patterns in cancer tissues can be interpreted as an indication that clinical cancer tissue is not a single clone but a heterogeneous population of cancer cells with various mutation patterns [37]. In this way, an attempt to determine the clinicopathological factors by analyzing CDO1 with immunostaining before treatment may be unsuitable when using localized tissue biopsy specimens. Quantitative values using TaqMeth V may be more useful for clinical application.

We then attempted to clarify the clinicopathological factors associated with *CDO1* methylation in primary CRC. *CDO1* methylation in CRC tissue was weakly influenced by the patient’s age. This finding was similarly obtained in adenoma (*p* = 0.06, R^2^ = 0.039) and NAM (*p* = 0.03, R^2^ = 0.046). This result was consistent with previous studies reporting that gene methylation increases with age [38]. Therefore, *CDO1* methylation can also be regarded as an age-related change. *CDO1* methylation was also significantly related to histological type, tumor diameter, liver metastasis, pT, Dukes classification, and v. This result clinically confirmed a previous study reporting that CDO1 participates in tumor cell growth, cell migration, invasion, and colony formation [39]. Consistent with this observation, using an anchorage-independent colony formation assay, this study showed that cell proliferation was suppressed following transfection of *CDO1* into a CRC cell line. Thus, our basic experiments confirmed the linkage between *CDO1* methylation abnormalities and the clinicopathological background. In addition, TaqMeth V was significantly higher in the differentiated type. This finding suggests that *CDO1* methylation may accumulate with tumor progression of CRC.

Although the pStage was an independent prognostic factor in multivariate analysis, when the relationship between *CDO1* methylation and prognostic outcomes was analyzed, separate optimal cut-off values of *CDO1* TaqMeth V were obtained for OS and RFS, and *CDO1* methylation was related to both prognostic outcomes. Such prognostic associations with *CDO1* methylation have been previously reported in various histological types of cancers such as renal clear-cell carcinoma, breast adenocarcinoma, and esophageal squamous cell carcinoma [10,18, 19]. The results of our study again for the first time suggested that *CDO1* methylation may be involved in the malignant progression of CRC.

In the 1950s and 1960s, the ACS theory was proposed in contrast to the de novo carcinogenesis theory as the major oncogenic pathway of CRC. However, in the 1970s, Morson et al. strongly advocated for the ACS theory, using the expression “polyp-cancer sequence,” and proposed that most CRCs are derived from adenomas [3]. Fearon [40] and Vogelstein [41] et al. proposed that multiple genetic abnormalities, such as those involving *adenomatosis polyposis coli*, cause the morphological change from adenoma to cancer. Subsequently, various genetic abnormalities associated with CRC have been identified. In recent years, next-generation sequencing of the genome in CRC has identified new CRC genes and demonstrated advanced genetic heterogeneity of tumor tissue and complex genetic abnormalities involved in carcinogenesis [42]. However, no report has described the relationship between epigenetic alterations and ACS.

Although *CDO1* methylation abnormalities involved in carcinogenesis have been reported, the *CDO1* oncogenic relevance during tumor progression remains unclear. We therefore compared the degree of aberrant methylation among NAM, adenoma, and cancerous tissue.

We measured TaqMeth V of adenomas classified into three stages (mild, moderate, and severe) on the basis of cellular atypia and structural atypia. TaqMeth V increased with advancing atypia (NAM < adenoma < cancerous tissue) (*p* < 0.0001). An important finding was that TaqMeth V differed significantly between NAM and mild atypia. These results suggested that aberrant methylation of *CDO1* is involved in the development of adenoma. Furthermore, the fact that TaqMeth V also differed significantly between low-grade adenoma and high-grade adenoma suggested that *CDO1* methylation contributes to the growth of adenoma and an increased grade of atypia. Although TaqMeth V did not differ significantly between high-grade adenoma and cancerous tissue, this finding reflects the difficulties in the clinical diagnosis of high-grade adenoma and cancer and the differential diagnosis of borderline lesions in clinical practice. However, because *CDO1* methylation was significantly higher in cancer with liver metastasis than in high-grade adenoma and cancer with no liver metastasis, *CDO1* methylation likely contributes to tumor cell migration and invasion.

*SEPT9* is a clinically applied methylation abnormality gene that has been established for CRC [29, 30]. Therefore, when comparing *CDO1* and *SEPT9*, methylation abnormalities tend to accumulate together with an increase in atypism of colorectal tissue. The AUC was 0.96 for *SEPT9* for calculating the cut-off value using the ROC curve that distinguishes between NAM and cancerous tissue. In addition, methylation increased with ACS. However, the carcinogenic process in which the methylation was significantly higher was slightly different in both cases. Methylation of *SEPT9* was increased during the course of adenoma-carcinoma, rather than gradually increasing during adenoma atypical progression. When cancer occurs, methylation of *SEPT9* does not change even if metastasis occurs. This result was different from that of *CDO1*, which increased with adenoma atypicality. In other words, these observations suggest that methylation of *SEPT9* is superior as a marker of cancer, and methylation of *CDO1* can be detected with high frequency in some types of adenoma. The combination of *CDO1* and *SEPT9* methylation could be a very effective tool for the detection of CRC or adenoma.

Although we could not conclude that accumulation of promoter DNA methylation of *CDO1* is a cause or a result of tumor progression, many reports have described the functional involvement of *CDO1* in cancer progression [15, 39, 43]. Because *CDO1* expression is inhibited by methylation in cancer cells, the production of glutathione is increased and resistance to reactive oxygen species is enhanced [43]. In addition, in esophageal cancer cell lines, forced expression of *CDO1* reduces tumor cell proliferation, cell migration, invasion, and colony formation [39]. In HBV-related HCC, the degree of *CDO1* methylation increases with malignant transformation (chronic hepatitis < cirrhosis < hepatocellular carcinoma) [15]. In this study, we elucidated that *CDO1* promoter DNA methylation accumulates along with tumor progression, and the highest level of *CDO1* promoter DNA methylation was observed in CRC with liver metastasis. Interestingly, venous invasion was also marginally associated with *CDO1* methylation in primary CRC tissues.

As mentioned above, our study suggested that aberrant methylation of *CDO1* is involved in the development and growth of adenoma, progression of atypia, oncogenic transformation, invasion, and metastasis. Namely, *CDO1* methylation induces cellular atypia, and the increased accumulation of *CDO1* methylation generates CRC. Not all specimens in our study were obtained from patients who received preoperative therapy. Our results are considered extremely important with respect to the clinicopathological features and involvement of *CDO1* in carcinogenesis in patients with CRC. Moreover, this report was able to provide new evidence that supports the ACS theory, which has been claimed as the main oncogenic pathway for CRC.

DNA methylation is a stable modification and is therefore expected to be a useful marker for the early detection of disease. DNA methylation accumulates in a tissue-specific manner [44, 45]. To date, studies evaluating abnormal *CDO1* methylation with the use of plasma obtained from patients with CRC have been performed [23], but the results have not yet been put into practical use. The detection rate of methylation abnormalities of *CDO1* in the plasma of patients with CRC is 20%, and thus, using plasma is difficult. Unlike *SEPT9*, clinical application of *CDO1* is considered to require approaches from specimens other than plasma. When excised samples were used, methylation abnormalities of *CDO1* could be distinguished from normal mucosa at the stage of adenoma as well as cancer tissue. This result seems to suggest that difficulties with detection of methylation abnormalities of *CDO1* in plasma for clinical application may be overcome by using, for example, a fecal specimen.

Because CRC has continued to be highly morbid worldwide, to avoid poor prognosis, early detection is essential. Although fecal occult blood testing is performed for CRC screening, progression of cancer caused by false-negatives occurs. We are planning to investigate biomarkers for the early detection of CRC by utilizing the differences in aberrant methylation among NAM, adenoma, and cancerous tissue demonstrated in the present study.

## Supporting information

S1 Table Sequences of primers and annealing temperatures for *CDO1*, *SEPT9*, and *β-actin* used in Q-MSP and RT-PCR. (XLSX)

S2 Table Univariate and multivariate prognostic analysis of clinicopathological factors for RFS in CRC (pStage 0-III). (XLSX)

S1 Fig ROC curve for *SEPT9* for distinguishing between tissue types. A: ROC curve for *SEPT9* methylation for distinguishing between cancer tissue and NAM. When the cut-off value was 0.06, the AUC was 0.96, sensitivity was 94%, and specificity was 95%. B: ROC curve for distinguishing between low-grade adenoma and NAM. C: ROC curve for distinguishing between low-grade adenoma and high-grade adenoma.
(TIF)

S2 Fig Verification of control cells in the analysis of methylation abnormalities in *SEPT9*. A: Results of Q-MSP of DLD1 and HepG2 cells. The mean TaqMeth V of DLD1 cells was 4.9, and that of HepG2 cells was 0. B: Primer creation area for bisulfite sequencing, including primers and known probes for Q-MSP of *SEPT9* [30]. Primers for bisulfite sequencing included 24 CGs, which are numbered in order. C: Cloned PCR products from DLD1 and HepG2 cells. White and black circles denote unmethylated and methylated CpG sites, respectively. The proportion of methylation was 99.2% in DLD1 cells and 0% in HepG2 cells. D: Results of Q-MSP of DLD1 cells. Serial dilutions of up to 1 × 10^−3^ resulted in amplification, and a calibration curve could be created.
(TIF)

S3 Fig Analysis of *SEPT9* TaqMeth V derived from NAM, adenoma, and cancerous tissue. A: Adenoma is classified into three categories: mild atypia, moderate atypia, and severe atypia. B: Adenomas were divided into low-grade adenoma and high-grade adenoma.
(TIF)
