# Supplementary figures and images for: Cysteine dioxygenase type 1 (CDO1) gene promoter methylation during the adenoma-carcinoma sequence in colorectal cancer
Source: PLoS One. 2018 May 10;13(5):e0194785. doi: 10.1371/journal.pone.0194785 (PMC5944981; doi:10.1371/journal.pone.0194785)

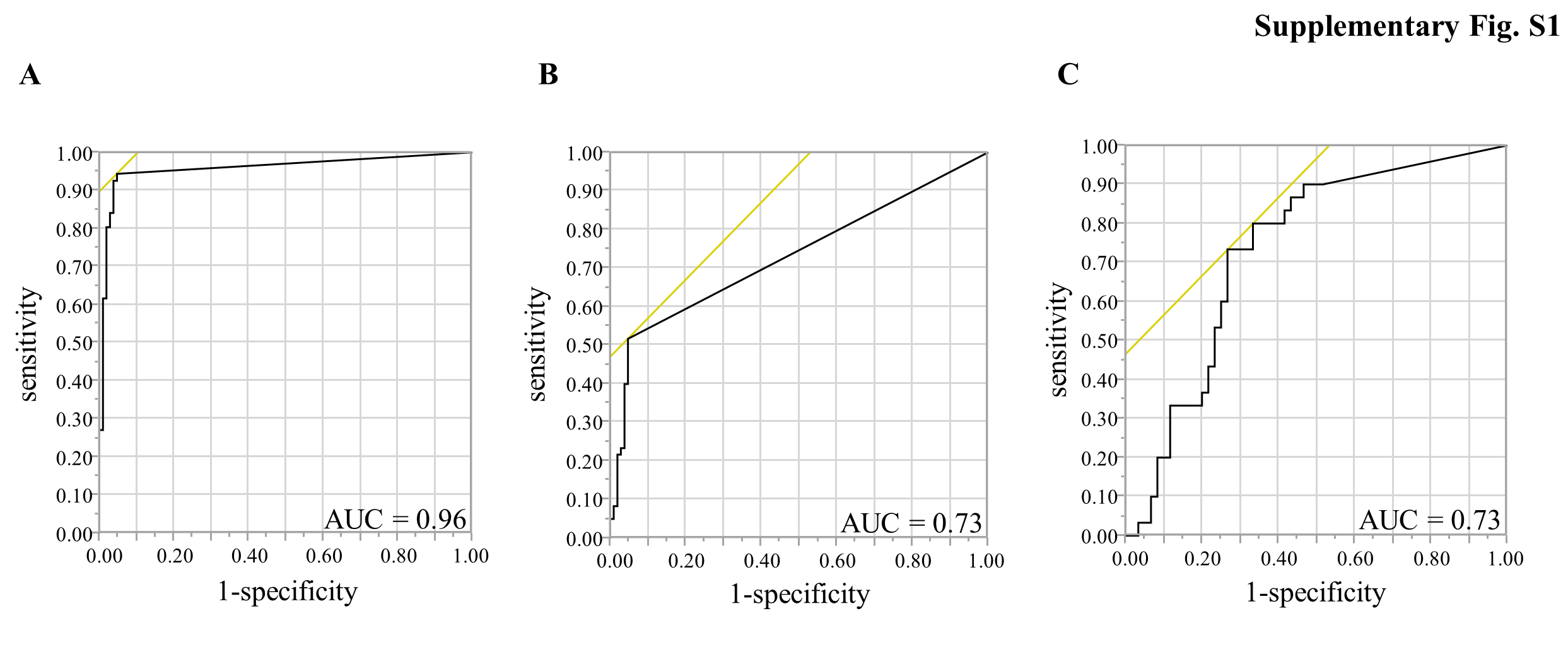

Supplement: S1 Fig — A: ROC curve for SEPT9 methylation for distinguishing between cancer tissue and NAM. When the cut-off value was 0.06, the AUC was 0.96, sensitivity was 94%, and specificity was 95%. B: ROC curve for distinguishing between low-grade adenoma and NAM. C: ROC curve for distinguishing between low-grade adenoma and high-grade adenoma. (TIF) [file pone.0194785.s003.tif]

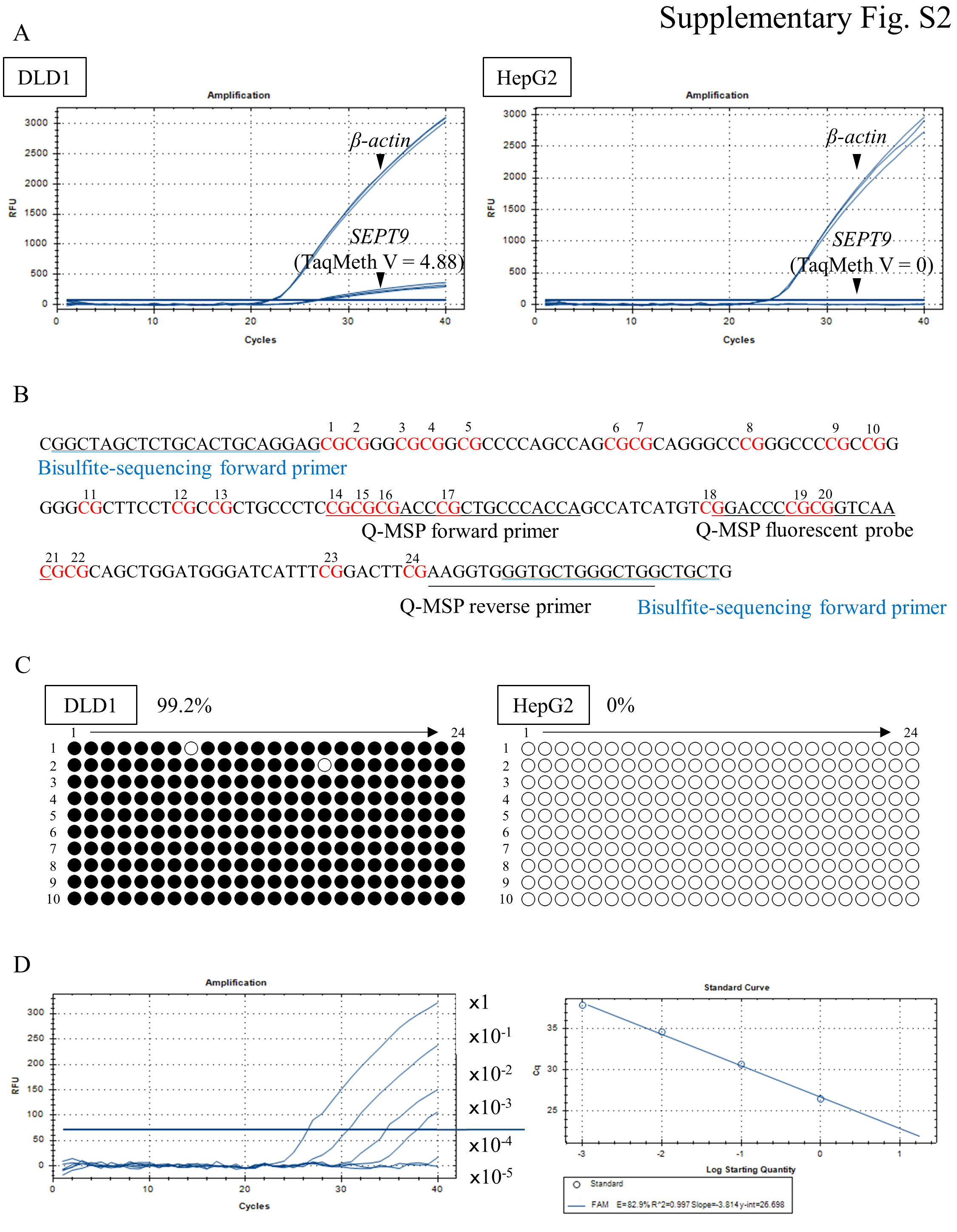

Supplement: S2 Fig — A: Results of Q-MSP of DLD1 and HepG2 cells. The mean TaqMeth V of DLD1 cells was 4.9, and that of HepG2 cells was 0. B: Primer creation area for bisulfite sequencing, including primers and known probes for Q-MSP of SEPT9 [30]. Primers for bisulfite sequencing included 24 CGs, which are numbered in order. C: Cloned PCR products from DLD1 and HepG2 cells. White and black circles denote unmethylated and methylated CpG sites, respectively. The proportion of methylation was 99.2% in DLD1 cells and 0% in HepG2 cells. D: Results of Q-MSP of DLD1 cells. Serial dilutions of up to 1 × 10^−3^ resulted in amplification, and a calibration curve could be created. (TIF) [file pone.0194785.s004.tif]

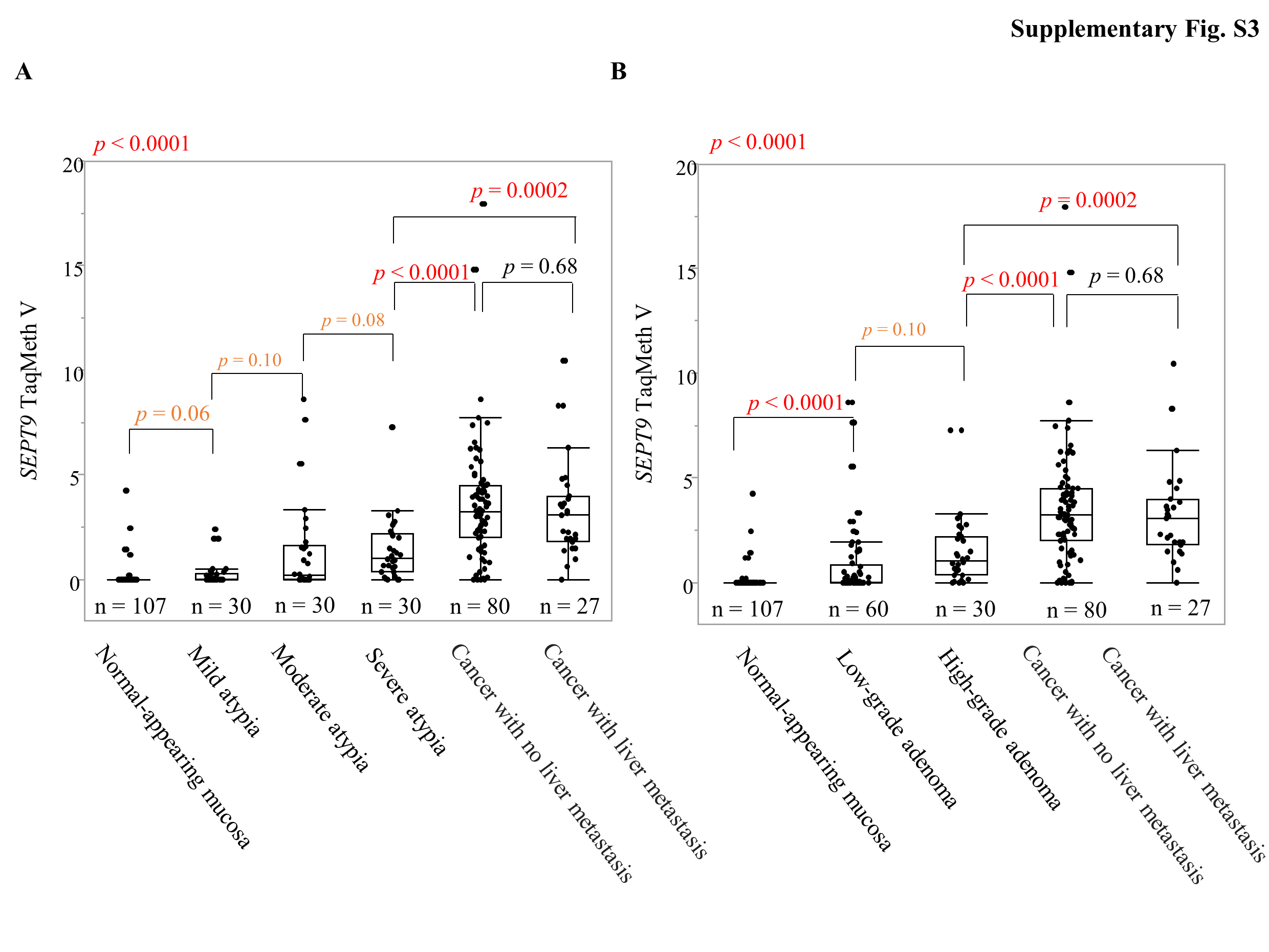

Supplement: S3 Fig — A: Adenoma is classified into three categories: mild atypia, moderate atypia, and severe atypia. B: Adenomas were divided into low-grade adenoma and high-grade adenoma. (TIF) [file pone.0194785.s005.tif]
